# Interlimb Coordination and Auditory—Motor Synchronization in Children with Developmental Coordination Disorder: Examining Antiphase Knee Movements with Auditory Metronomes While Seated

**DOI:** 10.3390/children11101195

**Published:** 2024-09-29

**Authors:** Mieke Goetschalckx, Lousin Moumdjian, Eugene Rameckers, Peter Feys

**Affiliations:** 1 REVAL Rehabilitation Research Center, Faculty of Rehabilitation Sciences, Hasselt University, Wetenschapspark 5-7, 3590 Diepenbeek, Belgium; lousin.moumdjian@uhasselt.be (L.M.); eugene.rameckers@uhasselt.be (E.R.); peter.feys@uhasselt.be (P.F.); 2 IPEM, Institute of Psychoacoustic and Electronic Music, Faculty of Art and Philosophy, Gent University, Miriam Makebaplein 1 B, 9000 Gent, Belgium; 3 Department of Rehabilitation Medicine, CAPHRI, FHML, Maastricht University, 6211 LK Maastricht, The Netherlands

**Keywords:** interlimb coordination, developmental coordination disorder, children, auditory–motor synchronization, motor timing

## Abstract

**Background:** Children with developmental coordination disorder (DCD) exhibit reduced interlimb coordination compared to typically developing children (TDC) during complex tasks like running, which requires dynamic postural control. However, the extent of interlimb coordination difficulties in DCD during tasks that demand minimal dynamic balance, such as self-paced and externally auditory-paced tasks, remains unclear. This study aimed to compare interlimb coordination and auditory–motor synchronization between children with DCD and TDC during a seated antiphase coordination task of the lower limbs, which has minimal postural control requirements. **Methods:** Twenty-one children with DCD and 22 TDC performed an antiphase knee flexion and extension task while seated, in three conditions (baseline silence, metronome discrete, and metronome continuous), for three minutes. The interlimb coordination, synchronization, and spatiotemporal movement parameters were analyzed using a mixed model analysis; **Results:** Children with DCD displayed less coordinated interlimb movements compared to TDC (*p* = 0.0140), which was the result of the greater variability in coordinating antiphase knee flexion–extension movements (*p* < 0.0001). No group differences in spatiotemporal movement parameters were observed. Children with DCD, compared to TDC, had a lower synchronization consistency to metronomes (*p* = 0.0155). Discrete metronomes enhanced interlimb coordination compared to the baseline silence condition (*p* = 0.0046); **Conclusions:** The study highlights an inferior interlimb coordination and auditory–motor synchronization in children with DCD compared to TDC. Implementing metronomes with a discrete temporal structure improved the interlimb coordination of both groups during the used fundamental seated interlimb coordination task, supporting theorical frameworks of event-based timing.

## 1. Introduction

Developmental coordination disorder (DCD) is a neurodevelopmental disorder that is characterized by uncoordinated motor performance of daily activities, ensuring that these deficits are not better explained by other neurological or medical conditions [1,2]. DCD is heterogeneous in its severity and presentation [2]. Individuals with DCD may exhibit marked difficulties in, but are not limited to, gross and fine motor coordination, postural control, motor planning, executive function, sensory–perceptual processing, dual tasking, timing, and coordination, as well as the acquisition and transfer of new motor skills [3,4,5].

As the diagnostic term implies, the key characteristic of children with DCD is impairments in coordination. Coordination can be defined as the effective control of the different degrees of freedom into a kinematic coupling or synergy formation that organizes a movement pattern to ensure stability under environmental demands to achieve a specific goal [6,7]. Although the underlying mechanisms and etiology of coordination deficits in DCD remain unclear, previous research has proposed a variety of hypotheses [2,3,8]. From a dynamical system approach, coordination emerges from the dynamical interaction between individual, task, and environmental constraints [4]. At the individual level, a variety of factors have been identified to constraint motor coordination in DCD. Impairments encompass, but are not limited to, the internal model, postural control, motor learning (automatization), executive function, sensory–perceptual processing, dual tasking, rhythmic coordination, and timing [4,5]. Additionally, coordination deficits in DCD are influenced by the nature and complexity of the task. Impairments are generally more prominent if the task require great endpoint precision, high complexity, advanced planning, high velocity, novel (non-familiar) tasks, and fast adaptations [3,4,5]. Lastly, environmental such as surface characteristics and a crowded surrounding may impact coordination [9,10,11].

Previous research examining coordination in children with DCD highlights the potential differences in inter- and intralimb coordination between children with DCD and typically developing children (TDC) across a variety of tasks, including bilateral finger tapping [12], ball catching [13], treadmill walking [14], and overground running [15]. Specifically, results showed that children with DCD exhibited more variability in their inter- and intralimb coordination across self-paced coordination tasks. It is important to note that the reported differences in coordination between children with and without DCD in the abovementioned articles may be partially attributed to the higher levels of physical activity, practice, dynamical postural control, familiarity, and automatization in TDC compared to children with DCD. Specifically, the lower levels of physical activity, familiarity, or automatization in DCD [16,17] may limit their opportunities to master fundamental motor skills, such as running and catching a ball [18].

Therefore, to minimize the impact of automatization and familiarity, Wilmut et al. examined coordination in a novel coordination task [19]. The novel task was a pedalo task, in which children needed to step onto a pedalo and pedal at their self-selected speed while maintaining an upright position, without stepping off or falling. Results confirmed that coordination pattern differ among children with and without DCD, even in a novel task that was not familiar for TDC. Although the aim of the new pedalo task was to examine coordination within a novel context, the authors noted that the TDC group had greater experience with tasks requiring balance. Additionally, the pedalo task requires a high degree of dynamic balance and control of multiple degrees of freedom to remain stable, which can be particularly challenging for children with DCD [20]. Research indicates that postural control difficulties are highly prevalent in DCD, affecting up to 87% [20,21]. As described above, previous research assessed the interlimb coordination of the lower limbs during tasks requiring postural control, such as walking [14,22], running [15], and a pedalo task [19], consistently reporting coordination differences between participants with and without DCD. Notably, these coordination differences were more pronounced during running compared to walking in children with DCD [15]. Running may be considered as more complex due to the absence of the double-support phase, which increases the demand for postural control, highlighting the possible impact of dynamical postural control on coordination in DCD. However, it remains unclear whether the observed coordination differences reported earlier are due to coordination (timing) issues [23] or secondary to postural control deficiencies [20,21]. Therefore, eliminating the need for postural control when assessing interlimb coordination of the lower limbs is essential in order to determine whether the coordination differences persist in tasks that require minimal dynamic postural control. This study therefore expanded previous research by minimizing the need for postural control with the aim to assess more fundamentally the interlimb coordination of the lower limbs.

Therefore, this study aimed to implement a coordination task designed to evaluate the interlimb coordination of the lower limbs, minimizing the need for dynamic postural control. The experimental task of antiphase knee movements while seated was specifically chosen to mimic as much as possible the antiphase movements of the lower limbs while walking, with minimal postural control requirement and degrees of freedom to control. A similar task has already been applied to assess interlimb coordination in other populations, including adults, the elderly, and persons with multiple sclerosis and Parkinson’s disease [24,25,26,27,28]. It is proposed that this approach may provide insights of fundamental understanding of interlimb coordination in children with DCD.

Although this was the first study to assess lower limb interlimb coordination in children with DCD using the seated antiphase knee movement task, it was hypothesized that interlimb coordination in DCD would be less stable than in TDC, even in a task with minimal postural control demands. This expectation aligns with the proposed hypothesis of a core timing deficit in DCD, as suggested by Trainor et al. [23]. Additionally, a key aim of this study was to examine auditory–motor synchronization and its effect on interlimb coordination. To explore this, an auditory–motor synchronization paradigm was included where participants were asked to align and couple their motor rhythm of antiphase knee movements with an external auditory rhythm (metronome beats). This paradigm has already been used in other populations [24] and was specifically chosen to assess how the dynamical interaction between individual, task and environment affects coordination [4]. Specifically, the rhythmic auditory beats may serve as a ‘gateway’ to the motor system by providing a temporal structure that synchronizes with movements, aiding alignment, and thereby enhancing coordination and motor performance [29,30,31,32,33]. Continuous auditory structures (e.g., enveloped metronome or music) are thought to promote continuous movements, such as more natural movement during walking [33,34]. Conversely, discrete auditory rhythms, marked by clear amplitude changes similar to an isochronous discrete metronome, may facilitate more synchronization with discrete movements [35].

It was hypothesized that children with DCD would have a lower synchronization consistency than TDC, irrespective of the metronome structure [23,32,36,37,38,39]. Additionally, it was hypothesized that synchronization and coordination during the seated interlimb coordination task would benefit more from the discrete metronome compared to the enveloped metronome, given the more discrete nature of the movement during the task.

This study contributes to our understanding of interlimb coordination and auditory–motor synchronization in children with DCD by using a seated antiphase knee movement task that minimizes postural demands. Isolating coordination from postural control allows for a more fundamental assessment of underlying motor timing mechanisms [23]. The findings from this research could offer important insights into the nature of coordination difficulties in DCD and contribute to developing more targeted interventions for children with DCD.

## 2. Materials and Methods

### 2.1. Participants

Participants aged from 8 to 12 years old were recruited through physiotherapists, sports centres, and schools by using flyers and social media. Participants were eligible to participate if they (a) were aged between eight and twelve, and (b) were typically developing or had a formal diagnosis or high probability of having DCD. The diagnosis of children with a formal diagnosis of DCD needed to be provided by an interdisciplinary specialized team of healthcare providers, following the international guidelines of DCD [2]. Participants were classified into either the DCD group (diagnosed or probable DCD) or TDC group, following the Diagnostic and Statistical Manual of Mental Disorders (DSM-V) criteria [1,2]. The participants were assessed by the main researcher (MG), a physiotherapist with specialization in paediatric physiotherapy, to check if the participants could be classified in the DCD group, meeting the DSMV criteria for DCD. These included (a) significant motor deficits, assessed by the movement assessment battery for children second edition (m-ABC2) [40] (participants with a subdomain score in the lower 5th percentile or a total score in the lower 16th percentile), (b) that these motor deficits negatively influenced activities of daily life, verified by the parent-reported Developmental Coordination Disorder Questionnaire (DCD-Q, Dutch translation CVO) [41], (c) that the onset of their motor symptoms was in childhood, verified using a parent-reported general health questionnaire, and d) that these motor deficits were not due to existing neurological, musculoskeletal, intellectual, or genetic disability, verified using a parent-reported general health questionnaire. Children were categorized in the TDC group if (a) the total percentile score on the m-ABC2 was in the upper 25th percentile, (b) the parents did not report a negative influence of motor impairments in activities of daily life, verified by the parent-reported Developmental Coordination Disorder Questionnaire (DCD-Q, Dutch translation CVO), and (c) they had no neurological, orthopaedical, cardiorespiratory, or intellectual impairment or diagnosis that could affect their motor abilities, using a parent-reported health questionnaire.

Participants were excluded if (a) they had behavioural difficulties that interfered significantly with testing and (b) they could not be categorized in the DCD, nor the TDC group given that they did not align with the abovementioned DSM-V criteria, thereby preventing clear categorization into either the DCD or TDC groups.

### 2.2. Study Design and Procedure

This cross-sectional case–control design was approved by the Medical Ethical Committee of the University of Hasselt (B1152020000009) on 18 August 2020. The larger cross-sectional study aimed to assess interlimb coordination and auditory–motor synchronization in children with DCD during three different tasks (walking, running, and antiphase knee flexion and extension while seated). This manuscript reports the results of the antiphase knee flexion and extension task. The research was conducted according to the Declaration of Helsinki and was registered in the clinical trials.gov registry (NCT04891562). Informed consent was signed by the parents of the participants.

The study consisted of two sessions, conducted on two separated days to minimize the impact of fatigue. Within the first session, descriptive demographic information, motor performance, and rhythm and melody perception were collected in a randomized order, using a computer-generated number randomizer (see Section 2.2.1, Session 1: Descriptive measures). The experimental paradigm (see Section 2.2.2, Session 2: Experimental paradigm) and executive functioning tests (see Section 2.2.1, Session 1: Descriptive measures) were performed during the second session in a randomized order. The first and second session took, respectively, 90 and 60 min, including rest periods.

#### 2.2.1. Session 1: Descriptive Measures

A parent-reported questionnaire was used to collect demographic information, including age, gender, early motor development, medical history, and participation in organized sports. Leg dominance was determined by asking the question, “Which leg do you use to kick a ball?”. Leg length of the dominant leg of the child was determined by measuring the distance between the spina iliaca anterior superior and the medial malleolus. The m-ABC2 test was used to assess gross and fine motor function, including manual dexterity, aiming and catching, and static and dynamic balance [40]. The m-ABC is a standardized and norm-referenced test that demonstrates good to excellent reliability, along with fair to good validity [42]. A total percentile score in the lower 16th percentile or a percentile score in the lower 5th percentile in any subdomain is indicative of being ‘likely to experience motor problems’ [40]. The second version of the Balance Evaluation Systems test for children (Kids BESTest) was performed to evaluate postural control more comprehensively. The second version of the Kids BESTest includes six domains: biomechanical constraints, stability limits and verticality, transitions/anticipatory, reactive, sensory orientation, and stability in gait [43]. Each item is assessed using a 4-point ordinal rating scale ranging from 0 (unable to perform independently) to 3 (normal performance); therefore, a higher total or domain score indicates a better performance. The total and domain scores of the full Kids BESTest have excellent to good reliability in school-aged children [44,45,46]. Executive functioning, namely, behavioral inhibition and working memory, was, respectively, assessed through an auditory Go-no/Go task [47] and the digit span (forwards and backwards) [48]. During the digit span, children were asked to listen to a sequence of random digits and repeat the sequence either forward or backward. To ensure comprehension, two practice trials with a sequence of two digits were conducted before the test. If the child correctly recalled both sequences, the test proceeded with an additional digit added to the sequence. Testing continued until the child failed to perfectly recall both trials of the same length. For both forward and backward digit spans, the score was the total number of correctly recalled sequences, with a higher score indicating better performance [48,49]. The Go-no/Go task was developed to assess behavioral inhibition in children with minimal working memory demands [47]. The test included 60 trials, with 75% being Go trials (sound of a dog) and 25% being no/Go trials (sound of a ringing bell). Children were instructed to press as quickly as possible the spacebar on a laptop when a Go stimulus appeared and to refrain from pressing when a no/Go stimulus appeared. The test measured mean reaction time, commission errors, and omission errors. Five practice trials were administered to ensure the children understood the task instructions. Lastly, rhythm and melody perception were assessed by two components (rhythm and melody) of the short version of the Montreal Battery of Evaluation of Musical Abilities (MBEMA-s) [50]. Both the melody and rhythm sections of the MBEMA-s consist of two practice trials and 20 test trials. Each trial presents two pairs of melodies or rhythms, and participants were asked to judge whether the pairs are similar or different. A higher score on the MBEMA-s reflects better performance, with a maximum of 20 points per subtest. A combined global score, representing the percentage of correct answers from both subtests, can be calculated, with a higher global score indicating better overall performance. All descriptive tests were assessed in a randomized order. The order of the descriptive tests was randomized by a computer-generated number randomizer.

#### 2.2.2. Session 2: Experimental Paradigm

**Figure 1:**
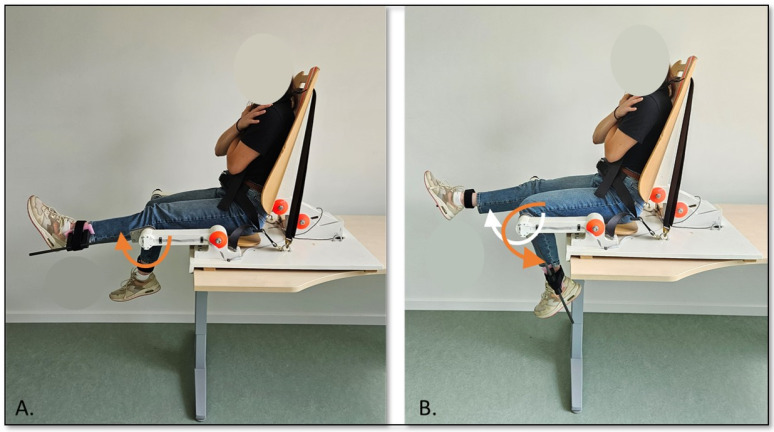
Set-up of the seated rhythmical interlimb coordination task: (**A**) extension of the left knee (orange arrow) while the right knee is flexed; and (**B**) flexion of the left knee (orange arrow), while the right knee is extended (white arrow).

The experimental paradigm was developed to assess antiphase interlimb coordination of the lower limbs. Participants were seated on a custom-made chair to which two levers were attached, allowing flexion and extension at the knee joint (see Figure 1). The levers were attached above the lateral malleoli by using a Velcro^®^ strip to allow the lower legs to perform knee flexion and extension movements. The lateral joint line of the knee was aligned with the axis of rotation of the levers by adapting the height of the axis of rotation of the levers and the back of the apparatus in anterior–posterior direction. To ensure stable posture, a four-point belt was used to strap the trunk of the participants in a comfortable position.

**Figure 2:**
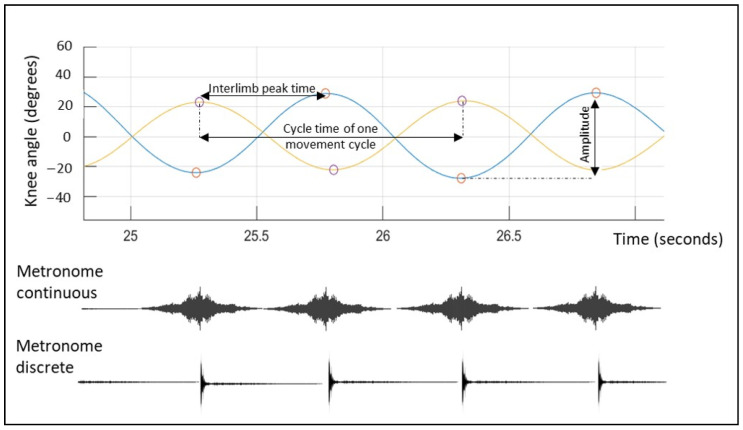
Graphical representation of the antiphase knee flexion and extension movements plotted against the two different temporal structures of the metronomes. The left (orange) and right (blue) knee movements in the sagittal plane are plotted (y-axis) over time (x-axis). Movement amplitude is expressed as the intralimb peak-to-peak signal for each individual movement cycle. Movement frequency is calculated as the amount of movement cycles in one minute. Moreover, the temporal structure of the two different metronomes (continuous and discrete) are plotted against the movement cycles.

After adjusting the apparatus to fit each child’s height and leg length, the children were familiarized with the task and asked to perform “seated walking”, which involves left-right antiphase flexion and extension of the knees so that one leg is up while the other is down. They were instructed to perform the task at their own preferred tempo and movement amplitude. A familiarization trial was conducted to ensure they understood the instructions. Following familiarization, a baseline recording of the experimental task was conducted for three minutes. This baseline recording was performed without auditory pacing the tempo (baseline silent condition) to capture their comfortable movement frequency. After a rest period of minimally two minutes, the participant performed the task with the instruction to couple and synchronize their leg movements to the beats of the auditory metronome conditions for three minutes. The instruction was as follows: “Repeat the previous task, but this time synchronize the movement tempo of your legs with the beats of the auditory metronomes. Ensure that at each beat, one leg is up and the other leg is down”. The metronome tempo was individually adjusted to the comfortable movement frequency during the baseline silent condition. A familiarization period proceeded the test performance to ensure the child understood the instruction. Two different isochronous metronome structures were used, namely, a metronome with an enveloped temporal structure (referred as the continuous metronome in the following text) and a discrete metronome [51]. The temporal structure of the continuous metronome followed a discrete isochronous pattern, yet entailed a continuous sinusoidal-like auditory structure, mimicking the temporal structure of music, while the temporal structure of the discrete metronome followed a discrete isochronous pattern with short distinct pulses indicating every beat. No differences were present between the metronome conditions in terms of pitch, or the interval structure in terms of tempo or phase. The order of the metronome conditions was randomized by a computer-generated number randomizer. Figure 2 provides a visual illustration of the two different metronome structures. The metronome conditions were performed in a randomized order.

### 2.3. Apparatus and Data Pre-Processing

At the axis of rotation of each lever-arm, a Seeeduino XIAO (CPU-ARM^®^ Cortex^®^-M0+(SAMD21G18), Kiwi Electronics B.V. Den Haag, The Netherlands) was mounted. The Seeeduino XIAO is a low-power Arduino microcontroller that allowed to capture the movements of the lever-arm, produced by the antiphase flexion–extension movements at the knee joints. MATLAB (R2019a) was used to process data and to derive cycle times (movement frequency), interlimb peak times, and movement amplitude (see Figure 2). Next, the metronome temporal structure was plotted against the timing of the antiphase flexion–extension movement in order to extract auditory–motor coupling parameters.

### 2.4. Primary Outcomes

Phase coordination Index (PCI): Interlimb coordination of the lower limbs during the antiphase flexion–extension movement was assessed by measuring the relative phase between the peak signals of the left and right legs (as illustrated in Figure 2, upper panel). The PCI was then calculated to quantify the accuracy and stability of this antiphase pattern. The PCI, originally developed to evaluate interlimb coordination during walking, has been used in previous studies to assess similar experimental protocols as those in this study [24,26,27,28,52]. The relative phase φ expresses the relative timing between the contralateral peak signals. Ideally, the relative phase φ should be 180° when performing antiphase coordination movements. The relative phase was determined by 360° × (interlimb peak time/cycle time). The absolute error of the relative phase to the ideal 180° was calculated with the following formula: PABSφ (%) = ((mean(ǀφ_i_ − 180ǀ))/180) × 100. The PABSφ expresses the accuracy of the relative phase. Moreover, coefficient of variation of the relative phases (CVφ) over time was calculated, using the following formula: CVφ (%) = (Standard deviation φ/mean φ) × 100. The CVφ expresses the stability in antiphase generation over the trial.

The PCI (%) takes into account both the accuracy of the relative phase (PABSφ) and the stability in antiphase generation (CVφ) by calculating the sum of the ABSφ and CVφ in percentage. The formula is as follows: PCI (%) = PABSφ + CVφ. A lower PCI reflects better phase control, achieved through a lower absolute error in the relative phases (PABSφ) and/or a lower coefficient of variation in the relative phases (CVφ) over time, indicating a better antiphase interlimb coordination.

### 2.5. Secondary Outcomes

#### 2.5.1. Spatial and Temporal Movement Parameters

The movement amplitude was expressed by the peak-to-peak amplitude, or the movement excursion for each individual movement cycle. The average movement amplitude over the task was calculated.

The movement frequency was expressed as the amount of movement cycles per minute for each leg. One movement cycle was defined between two successive peak extension positions. The average movement frequency over the task was calculated.

#### 2.5.2. Auditory–Motor Synchronization Parameters

Tempo matching was determined to assess the ability to match their movement frequency to the tempo of the metronomes. The following formula was used to assess tempo matching (%): (movement frequency/beats per minute) × 100. A score of 100% tempo matching indicates, on average, a perfect tempo matching.

Relative phase angle (rPA) and resultant vector length (RVL). The auditory–motor synchronization accuracy and consistency between the motor rhythm to the phase of the auditory rhythm during the metronome conditions was computed using circular statistics [53]. The relative phase angle (°) is a measure of accuracy in synchronization. It describes the timing of the peak extension position relative to the closest beat. A positive angle represents a peak extension position after the beat (reacting or lagging the beat), and a negative angle occurs when the peak extension position is before the closest beat (anticipating). The RVL expresses the consistency or stability of the relative phase angles over time, and is a measure with units ranging from zero to one [53]. A RVL closer to one represents a high coherence of relative phase angles, implying a high synchronization consistency. In contrast, a RVL of zero represents a multimodal distribution of the relative phase angles over time, indicating a lower synchronization consistency.

### 2.6. Statistical Analysis

Descriptive data were compared between groups using an independent t-test or nonparametric Wilcoxon/Kruskal–Wallis test for, respectively, normal and non-normal distributed continuous data. Normality was checked by a Shapiro–Wilk test. Categoric descriptive data were compared between groups by using the Fisher (exact) test.

Primary and secondary outcomes were analyzed by using a mixed model analysis of variances (ANOVA) with backwards modelling. This model included a random effect of participant IDs, main effects of Group (DCD and TDC) and Condition (silence, metronome discrete, and enveloped metronome), along with their interactions. For both primary and secondary outcomes, the normal distribution of the final model was checked using conditional residual plots. If a main or interaction effect was significant (at a significance level of α = 0.05), a post hoc Tukey test was performed, using Tukey–Kramer adjustment for multiple comparisons. All analyses were conducted using JMP Pro 17.0.0. Outliers were checked using quantile range outlier detection method in JMP Pro. No outliers were detected, and, therefore, all data were used for statistical analysis.

## 3. Results

### 3.1. Participants

**Figure 3:**
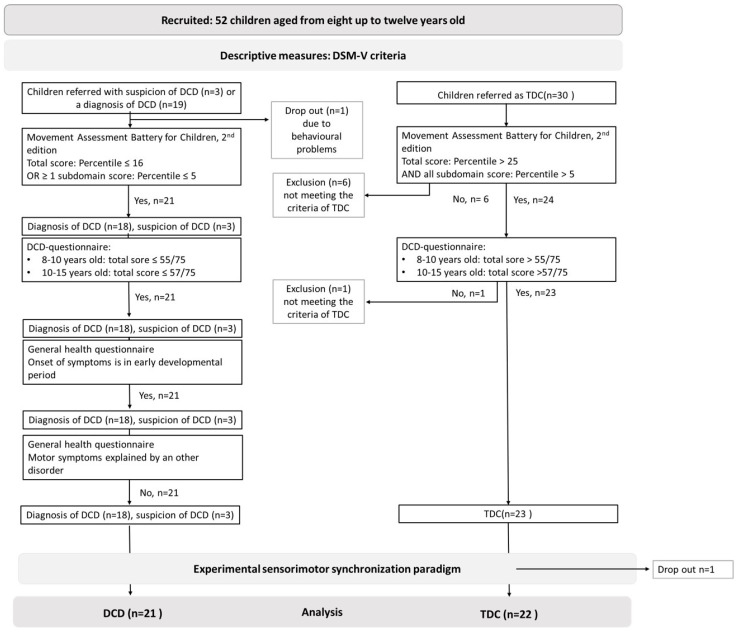
Flow chart of the participants. Abbreviations: developmental coordination disorder (DCD), number (*n*), and typically developing children (TDC).

Fifty-two children, aged between eight and twelve years old, were recruited. Among them, 30 were referred to as TDC, while the remaining 22 were referred to as either diagnosed with DCD (*n* = 19) or likely to have DCD (*n* = 3). After screening based on the study inclusion and exclusion criteria, 23 were categorized as TDC and 21 children as DCD. Children were excluded when: (a) they did not meet the selection criteria of the (probable) DCD, nor the TDC group (*n* = 7); (b) if the testing could not be completed due to behavioral problems (drop-out, *n* = 1); or (c) if they did not complete the full experimental protocol (*n* = 1, TDC). A flow chart of the participants can be found in Figure 3. Participant characteristics can be found in Table 1.

The DCD and TDC groups were similar in age, digit span scores, melody and rhythm perception (MBEMA-s), years of music lessons, and auditory Go-no/Go commission errors and reaction time. However, there were significant differences in gender distribution, with 81% boys in the DCD group compared to 41% in the TDC group. Additionally, children with DCD had significantly lower scores on the m-ABC2, KidsBESTest, and DCD-Q. Furthermore, children with DCD made significantly more omission errors during the auditory Go-no/Go, leading to fewer correct answers than those in the TDC group. Table 1 presents the sample descriptive information and the between-group results.

### 3.2. Primary Outcomes

Table 2 summarizes the mean and standard deviation of the primary outcomes during the experimental paradigm, including the statistical results.

Absolute error of the relative phase (PABSφ): No significant main group, condition, or interaction effects were observed.

Coefficient of variation of the relative phases (CVφ): A significant main effect of group [F(1,41) = 19.62, *p* < 0.0001] and condition [F(2,84) = 5.66, *p* = 0.0049] was observed. A post hoc multiple comparison revealed that, in all conditions, children with DCD have a significantly higher CVφ than TDC (*p* < 0.0001). Additionally, post hoc tests indicated that, in both groups, the CVφ is significantly lower in the metronome discrete (*p* = 0.0083) and metronome continuous (*p* = 0.0199) condition compared to the silence condition.

**Figure 4:**
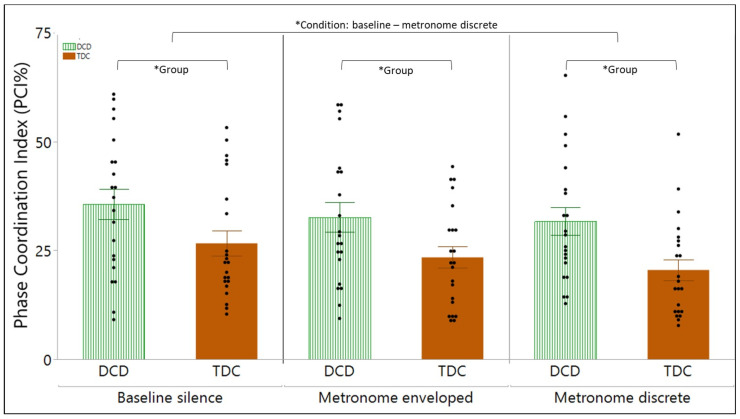
Significant main group and main condition effect on the phase coordination index (PCI). The main effect of group (* Group) indicates that children with developmental coordination disorder (DCD, green striped bar) have a significant higher PCI than typically developing children (TDC, orange bar), regardless of the condition (baseline, metronomes). The main effect of condition (* Condition) indicates that the PCI significantly differ between the baseline silence conditions and the metronomes discrete condition, regardless of the group (DCD and TDC). Bars represents mean and standard error.

Phase coordination index (PCI): A significant main effect of group [F(1,41) = 6.59, *p* = 0.0140], and a significant main effect of condition [F(2,84) = 5.41, *p* = 0.0061] was found. A post hoc multiple comparison revealed that, in all conditions, children with DCD have a significantly higher PCI than TDC (*p* < 0.0001). Additionally, post hoc tests indicated that, in both groups, the PCI is significantly lower in the metronome discrete (*p* = 0.0046) condition compared to the silence condition. Figure 4 visualizes the results of the PCI.

### 3.3. Secondary Outcomes

Movement amplitude: No significant main group, condition, or interaction effects were observed.

Movement frequency: A significant main effect of condition [F(2,84) = 4.89, *p* = 0.0098] was observed. A post hoc multiple comparison revealed a significant, albeit small difference, in the movement frequency between the baseline silence and the metronome continuous condition (*p* = 0.0070). Specifically, the movement frequency was 0.77 movements per minute lower in the metronome continuous condition compared to the baseline silence condition.

**Figure 5:**
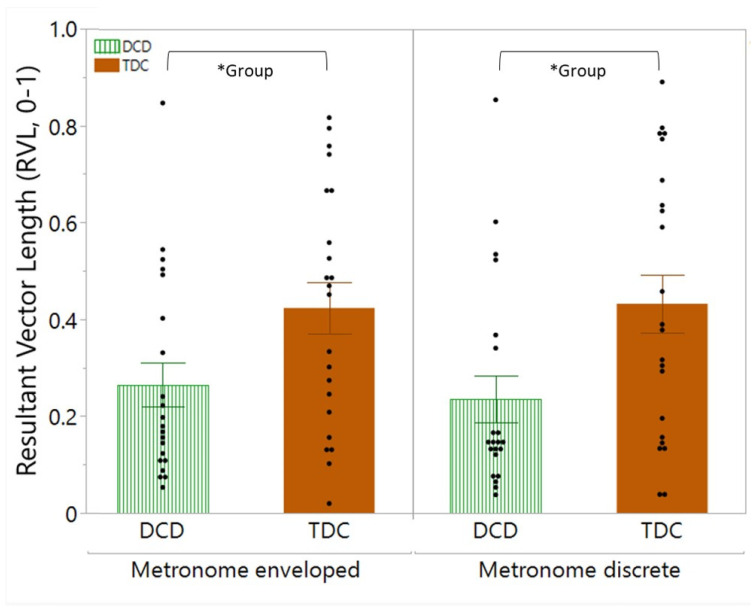
Significant main effect of group on the resultant vector length (RVL). The main effect of group (* Group) indicates that children with developmental coordination disorder (DCD, green striped bar) have a significant lower RVL than typically developing children (TDC, orange bar), regardless of the metronome condition. Bars represents mean and standard error.

Auditory–motor synchronization: A significant main effect of condition was observed for tempo matching [F(1,42) = 6.77, *p* = 0.0128]. Post hoc multiple comparisons indicated a small but significant difference in tempo matching between the metronome discrete and continuous condition (*p* = 0.0128, difference 0.78). Specifically, during the metronome continuous condition, the movement frequency was slightly lower than the pre-set metronome tempo, leading to a tempo matching value above 100%. Conversely, during the discrete metronome condition, the movement frequency was slightly higher than the pre-set metronome tempo, resulting in a tempo matching value slightly below 100%. In addition, a significant main effect of group [F(1,41) = 6.38, *p* = 0.0155] was observed for the resultant vector length (RVL). Post hoc comparisons revealed that the RVL was significantly lower in the DCD group compared to the TDC group in both metronome conditions (*p* = 0.0155) (see Figure 5). No significant main group, condition or interaction effect was found for the relative phase angle (rPA).

Table 3 summarizes the mean and standard deviation of the secondary outcomes during the experimental paradigm, including the statistical results.

## 4. Discussion

The primary aim of this study was to examine the interlimb coordination of the lower limbs during an antiphase knee flexion and extension movement while seated, thereby minimizing the need for dynamic postural control and reducing the degrees of freedom to control. Secondly, the study aimed to examine auditory–motor synchronization and its effect on interlimb coordination during the seated interlimb coordination task of the lower limbs. Additionally, the impact of the temporal structure (continuous versus discrete) of the auditory metronome on interlimb coordination and auditory–motor synchronization was investigated. The major findings of this study using a seated interlimb coordination task were that (a) children with DCD displayed less coordinated interlimb movements compared to TDC, indicated by a significantly higher phase coordination, which was attenuated by the greater variability (CVφ) in coordinating antiphase knee flexion–extension movements; (b) children with DCD, compared to TDC, had a lower synchronization consistency to metronomes, expressed by a lower RVL; and (c) incorporating metronomes (with a discrete temporal structure) assisted children with lowering PCI compared to the baseline silence recording, thereby promoting a more consistent antiphase interlimb coordination.

The main finding of the impaired interlimb coordination in DCD compared to TDC during the seated interlimb coordination task of the lower limbs confirms our hypothesis—namely, that children with DCD exhibit a higher variability in their phase coordination (CVφ), and, consequently, a higher PCI compared to TDC. Although clear between-group differences in interlimb coordination were observed, both groups performed the task with a similar movement amplitude and frequency. This result indicates that a similar task outcome (spatiotemporal movement parameters) was reached, although with different coordination patterns between children with and without DCD. Specifically, a higher average PCI was observed in children with DCD compared to TDC, regardless of the condition (baseline and metronomes). Our experimental paradigm was specifically designed to objectively quantify motor timing [23], while minimizing the need for dynamic postural control and reducing the degrees of freedom to control. Given that previous research assessed interlimb coordination of the lower limbs during tasks requiring postural control [14,15,19], they could not conclude if the observed differences in coordination were the result of postural control difficulties in DCD. The results of interlimb coordination in the present study, namely, a significantly higher PCI in DCD compared to TDC, indicated that children with DCD have difficulties in interlimb coordination in a task with minimal needs of postural control. Therefore, our results suggest a fundamental motor timing difficulty in DCD, which can be defined as the ability to accurately and consistently coordinate and perform inter- or intralimb movements [23,54]. This result is also supported by previous research, reporting differences in inter- or intralimb coordination between children with and without DCD [12,13,14,15,19]. The observed interlimb coordination differences in DCD compared to TDC may be due to the presence of atypical neural structures and functions within networks that support motor planning, coordination, and timing. These may include the altered white matter microstructure in the corpus callosum, sensorimotor, corticospinal, cortico-cerebellar, and frontoparietal pathways [55,56,57,58], and the cerebellum [3,59,60,61]. Evidence suggests a link between reduced structural integrity of the corpus callosum and poorer bimanual coordination [62], and, recently, the integrity of the corpus callosum has also been linked to interlimb coordination during overground walking in persons with neurological disorders, such as in persons with multiple sclerosis [63]. Additionally, the cerebellum plays a crucial role in the smooth coordination and timing of motor control [64,65]. Therefore, it may be valuable to explore various neural areas, including the corpus callosum, cerebellum, and functional networks, in relation to interlimb coordination to increase our fundamental understanding of interlimb coordination in DCD.

Although significant group differences were observed in PCI during our experimental paradigm, the reported PCI values during the baseline recording of the seated antiphase coordination task were larger on average (DCD 35.66%, TDC 26.64%) compared to reported average PCI values during overground walking (DCD 6.59, TDC 6.21) or running (DCD 8.33, TDC 5.07) in a similar population group [15]. An explanation of the relatively high PCI in this study could be mediated by the large variability in relative phases over time (CVφ). This might be due to the task novelty (non-familiar task) compared to walking and running, activities that most children perform every day. It is believed that a high variability of coordination patterns may indicate an initial exploration of potential solutions for successfully completing the motor goal of a novel task [66,67]. This might explain the relatively high CVφ, and, consequently, high PCI, in both DCD and TDC during the seated interlimb coordination task compared to walking or running. Although the task used in this study might be experienced as novel for children with DCD, dynamic postural control and the control of different degrees of freedom were limited compared to walking or running. Previous research suggested that task complexity might exaggerate interlimb coordination difficulties in DCD, which were more prominent during running compared to walking [15]. Running may be seen as more complex compared to walking, given the higher need for dynamical postural control and accurate timing [68], factors that may influence coordination in children with DCD [4]. It seems that task complexity is also an important factor to consider when assessing coordination in children with DCD. Therefore, it is important to consider both the novelty (i.e., familiarity) of the task and its complexity when assessing motor timing or coordination in children with DCD.

The second aim of this study was to examine auditory–motor synchronization and its effect on interlimb coordination. Our results revealed that children with DCD, compared to TDC, have a lower synchronization consistency, expressed by a lower RVL. However, both groups had on average a similar tempo matching ability and average relative phase angle. These results are in accordance with previous studies investigating auditory–motor synchronization in DCD during finger-tapping, walking, or running [12,32,36,69,70,71,72]. From a theoretical standpoint, auditory–motor synchronization is governed by the dynamic process of entrainment which involves the coupling of a motor rhythm to the auditory beats to reach a state of synchronization in phase and period [73]. Entrainment can be explained through an error-prediction minimization process [73,74], or, alternatively, through a dynamical process [75]. Within the used auditory–motor paradigm in this study, the error-prediction minimizing process explains entrainment by the process of minimizing timing differences between the peak movement amplitude and the auditory beats, so that, within time, the motor rhythm aligns with the auditory rhythm. Following this error-prediction minimization process, the observed difficulties in auditory–motor synchronization in DCD may be the result of an internal modelling deficit, or, in other words, a deficiency in generating or implementing predictive models [5,72,76], hampering the error-prediction minimizing process. On the other hand, within the dynamical systems theory, the motor and auditory systems are seen as two oscillatory systems which exert mutual forces on each other to reach a state of coupled dynamical systems. Therefore, the dynamical systems perspective attributes auditory–motor synchronization difficulties to the interaction between internal and external forces, with reduced temporal motor stability reflecting challenges in dynamic movement control [38,77].

Our results showed that metronomes with the discrete temporal structure enhanced interlimb coordination, specified by a decrease of 5% in PCI during the metronome discrete condition compared to the baseline silence condition. Previous research examining the impact of implementing metronomes on (interlimb) coordination during walking and running did not find a significant effect of metronomes on interlimb coordination [32]. This discrepancy may be the result of the different types of tasks and task complexity levels of walking/running and the present experimental task of antiphase knee flexion and extension. To elaborate, these results might be explained due to the timing mechanism underlying auditory–motor coupling with the discrete metronomes, specifically event-based (or explicit) timing [78,79]. The discrete metronome includes singular events, or sharp energy burst of the metronome intensity, indicating discrete moments in time. Event-based timing, linked to discontinuous movements, is believed to involve an internal timekeeper that represents explicit time intervals [79]. To elaborate, the clear extraction of the metronome beats likely aids in estimating the metronome tempo, as well as forming the error-prediction by comparing the timing of the beats with the movements. As a result, the clear discrete metronome beats may facilitate planning and execution of the movements by increasing the temporal stability of the antiphase flexion and extension movements in our experimental paradigm. In contrast, emergent-based (or implicit) timing is supposed to underly continuous movements (e.g., walking) where the motor rhythm emerges from the intrinsic dynamics of the effector system (e.g., oscillatory stiffness, and the spring-mass model during locomotion [80]). The emergent-based timing mechanisms reflects more the limit-cycle dynamics of the dynamical system where two oscillatory systems exert mutual forces on each other to reach a state of coupled dynamical systems. These frameworks of event-based and emergent-based timing address different aspects of perceptual-motor timing, and their relevance varies depending on the task demands, including the movement type within the task, reflecting more discontinuous (e.g., tapping) or continuous (e.g., cycling and walking) movements [35,78,79]. For example, previous research has shown that walking performance, assessed by spatiotemporal variability and gait dynamics, might indeed benefit more from auditory rhythms with a continuous temporal structure compared to discrete metronomes [32,33,34].

The different timing mechanisms underlying auditory–motor coupling with the discrete metronomes, specifically event-based (or explicit) timing, might also explain the significant, albeit small, difference in tempo matching and movement frequency between the two metronome conditions. Although both groups could almost perfectly match their average movement frequency with the metronome tempo, the movement frequency was slightly closer to the pre-set metronome tempo during the discrete metronome compared to the metronome envelop condition. The clear extraction of the discrete metronome beats likely aids in estimating the metronome tempo. In contrast, the gradual change in metronome amplitude might be more difficult for accurately extracting the metronome tempo. Given this gradual change, the metronome envelop might be perceived as slower, resulting in a lower movement frequency.

Several methodological considerations should be mentioned. A first methodological consideration is the envelope used to create a continuous temporal structure within a metronome rhythm. This implementation aimed to mimic the temporal structure of music. However, it is possible that the temporal envelope used in this study did not adequately reflect the structure of music, even though an effect consistent with the literature regarding the differential effect of discrete versus continuous (music) temporal structure on motor outcomes was observed [33,34,81]. A second methodological consideration is the relatively small sample size. Since this study is the first to examine the interlimb coordination (PCI) of the lower limbs during seated antiphase knee movements, an a priori sample size calculation could not be performed. In order to reflect on our sample size, we used G*Power to estimate the achieved power for the primary outcome PCI. The analysis revealed that our sample size of 21 in the DCD group and 22 in the TDC group ensured a power of 0.98 (β = 0.02) and an alpha value 0.05 for the main effect of group on PCI. We, therefore, suggest that our sample size of 21 in the DCD group and 22 in the TDC group was adequate to answer our main research questions and aims with sufficient statistical power. Lastly, we note the limited psychometric integrity of some demographic tests used in the study [49]. The aim of the expanded descriptive assessment was to have an elaborated view of the study sample characteristics due to the large heterogeneity in DCD [21]. However, it is proposed that individual factors, including executive functioning [82,83], musical experience [84,85], and auditory perception [86] may impact auditory–motor synchronization. Therefore, further research is recommended to explore the role of individual characteristics using sensitive and reliable tests on synchronization. Recommendations for further research also imply the use different metronome tempi, and phase and tempo shifts within the task to assess adaptability of the children. In this study, the children were asked to perform the antiphase knee flexion and extension movements at their preferred comfortable pace without defining the movement amplitude. The metronome tempo was set accordingly to the individual comfortable movement frequency. However, previous studies have stated that altered metronome tempi, higher or lower than the preferred motor frequency, might be even more challenging for children with DCD [36,37,69,71]. Therefore, further studies are recommended to include higher and lower metronome tempo to investigate interlimb coordination during a controlled task with challenging levels of complexity.

While the study primarily aimed to explore fundamental interlimb coordination and synchronization in children with DCD, the findings have potential rehabilitation applications. These benefits are twofold: First, there is a value for assessments that focus specifically on interlimb coordination in children with DCD. The fundamental coordination assessment used in this study could complement existing functional assessments, which are primarily capacity-based (e.g., Körperkoordinationtest für Kinder, and Bruininks–Oseretsky Test of Motor Proficiency-2) [87] and often include dynamic balance tasks. Adding this more fundamental assessment may provide a deeper understanding of a child’s coordination difficulties by incorporating tasks of varying complexity [4]. Second, integrating metronomes with discrete temporal structures into training programs may help improve consistent antiphase interlimb coordination. Further intervention studies are needed to the effect on interlimb coordination across different tasks.

## 5. Conclusions

Overall, the results are indicative of the presence of fundamental motor timing difficulty in DCD. The findings revealed an inferior interlimb coordination (motor timing), driven by a larger variability of antiphase coordination movements, in children with DCD compared to TDC during antiphase coordination knee flexion and extension movements of the knee. In addition, the results indicate that both groups can match their movement tempo to that of the metronome, yet with children with DCD doing so with a lower synchronization consistency than TDC. Lastly, implementing metronomes with a discrete temporal structure improved the interlimb coordination of both groups.

## Figures and Tables

**Table 1:** Descriptive characteristics (mean (standard deviation)) of the participants.

		DCD (*n* = 21)	TDC (*n* = 22)	*p*-Value
Age (years)		10.27 (1.53)	10.46 (1.20)	0.5514 ^a^
Body weight (kilograms)		36.85 (10.60)	36.12 (7.02)	0.7521 ^a^
Body length (centimetres)		143.16 (14.45)	145.17 (9.50)	0.5951 ^d^
Leg length (meters)		0.75 (0.08)	0.78 (0.07)	0.2015 ^d^
Gender	*n* (%boys)	17 (81%)	9 (41%)	**0.0122** ^b^
Comorbidity diagnosis	Total %	35%	0%	
	AD(H)D (*n*)	3		
	ASD (*n*)	3		
	CVI (*n*)	1		
	Learning disorder (*n*)	2		
DCDQ (/75)		35.14 (9.92)	70.55 (3.38)	**<0.0001** ^c^
m-ABC-2 (percentile 0–100)	Total	7.34 (10.20)	64.14 (19.45)	**<0.0001** ^c^
	Manual dexterity	11.00 (17.33)	60.36 (32.01)	**<0.0001** ^c^
	Aiming and catching	9.47 (13.31)	50.45 (24.52)	**<0.0001** ^c^
	Balance	19.85 (25.86)	60.73 (17.96)	**<0.0001** ^a^
Kids BESTest (0–100%)	Total	79.66 ± 8.15	93.99 ± 3.92	**<0.0001** ^c^
	Domain I	88.89 ± 11.80	97.88 ± 3.79	**<0.0001** ^a^
	Domain II	68.48 ± 11.71	83.98 ± 11.05	**<0.0001** ^a^
	Domain III	75.13 ± 16.63	96.21 ± 6.73	**<0.0001** ^c^
	Domain IV	84.39 ± 10.34	95.45 ± 6.33	**0.0003** ^a^
	Domain V	91.75 ± 8.14	99.09 ± 2.34	**0.0006** ^c^
	Domain VI	69.31 ± 17.37	91.31 ± 9.67	**<0.0001** ^c^
Digit span forwards (0–18)		7.52 (1.99)	6.77 (1.34)	0.3376 ^a^
Digit span backwards (0–16)		4.24 (1.41)	4.77 (1.38)	0.1823 ^a^
Auditory Go-no/go	Correct (0–60)	54.67 (6.92)	58.59 (2.09)	**0.0202** ^c^
	Omission errors (0–60)	3.05 (6.15)	0.18 (0.50)	**0.0458** ^c^
	Commission errors (0–60)	2.29 (2.78)	1.23 (1.77)	0.2636 ^a^
	Reaction time (ms)	817.14 (214.37)	803.68 (134.40)	0.8077 ^d^
Music lessons (years)		0.76 (1.48)	0.73 (1.32)	0.9510 ^a^
MBEMA-s total % (0–100%)		75.92 (13.08)	78.17 (12.17)	0.4548 ^a^

Data are mean (standard deviation). Between-group differences are reported with the corresponding *p*-value. Bold *p*-values are considered as significant using two-sided *p*-values < 0.05. Abbreviations: Attentional deficit (hyperactivity) disorder (AD(H)D), autism spectrum disorder (ASD), cerebral visual impairment (CVI), developmental coordination disorder (DCD), typically developing children (TDC), Developmental Coordination Disorder Questionnaire (DCDQ), Movement Assessment Battery—Second Edition (m-ABC-2), *n* = number, ^a^ Wilcoxon signed-rank test, ^b^ Fisher’s exact test, ^c^ Welch’s Test, ^d^ independent *t*-test.

**Table 2:** Results of interlimb coordination during the experimental paradigm.

Outcome	Condition	Group		Statistics					
		DCD (*n* = 21)	TDC (*n* = 22)	Backwards Mixed Model Analysis of Variances	Post Hoc Multiple Comparison: Tukey HSD All Pairwise Comparison, Tukey–Kramer Adjustment				
					Comparison	Difference	SE	t-Ratio	*p*-Value
PABSφ	Baseline silence	21.12 (11.97)	18.35 (12.00)	Not significant					
	Metronome discrete	19.08 (12.52)	13.32 (10.54)						
	Metronome continuous	20.24 (14.32)	15.70 (9.99)						
CVφ	Baseline silence	14.54 (5.60)	8.29 (3.52)	* Group [F(1,41) = 19.62, *p* < 0.0001] * Condition [F(2,84) = 5.66, *p* = 0.0049]	DCD–TDC	5.43	1.22	4.43	<0.0001
	Metronome discrete	12.58 (5.51)	7.18 (2.52)		Discrete–silence	−1.52	0.50	−3.06	0.0083
	Metronome continuous	12.35 (4.94)	7.71 (3.80)		Continuous–silence	−1.37	0.50	−2.75	0.0199
PCI	Baseline silence	35.66 (16.02)	26.64 (13.51)	* Group [F(1,41) = 6.59, *p* = 0.0140] * Condition [F(2,84) = 5.41, *p* = 0.0061]	DCD–TDC	9.79	3.81	2.57	0.0140
	Metronome discrete	31.66 (14.60)	20.50 (11.25)		Discrete–silence	−5.09	1.56	−3.26	0.0046
	Metronome continuous	32.59 (15.57)	23.41 (11.64)						

Data are mean (standard deviation). Results of the backward repeated mixed model analyses are reported by F(df) = F value, *p*-value. Significant results after post hoc multiple comparison with Tukey–Kramer adjustments are reported. Abbreviations: * statistical significant results of the backwards repeated mixed model analysis, absolute error of the relative phase (PABSφ), coefficient of variance of relative phases (CVφ), developmental coordination disorder (DCD), number of participants (*n*), phase coordination index (PCI), standard error (SE), and typically developing children (TDC).

**Table 3:** Results of secondary outcomes during the experimental paradigm.

Outcome	Condition	Group		Statistics					
		DCD (*n* = 21)	TDC (*n* = 22)	Backwards Mixed Model Analysis of Variances	Post Hoc Multiple Comparison: Tukey HSD All Pairwise Comparison, Tukey–Kramer Adjustment				
					Comparison	Difference	SE	t-Ratio	*p*-Value
Movement amplitude	Baseline silence	74.40 (18.15)	67.08 (19.14)	Not significant					
	Metronome discrete	73.07 (18.80)	65.85 (13.27)						
	Metronome continuous	71.25 (19.29)	68.79 (13.36)						
Movement frequency	Baseline silence	59.86 (4.70)	60.14 (4.20)	* Condition [F(2,84) = 4.89, *p* = 0.0098]	Continuous–silence	−0.77	0.25	−3.12	0.0070
	Metronome discrete	59.65 (4.49)	59.70 (3.80)						
	Metronome continuous	59.06 (4.37)	59.39 (3.66)						
Tempo matching	Metronome discrete	101.55 (5.29)	99.29 (2.72)	* Condition [F(1,42) = 6.77, *p* = 0.0128]	Discrete–continuous	0.78	0.30	2.60	0.0128
	Metronome continuous	100.50 (4.23)	98.77 (2.12)						
RVL	Metronome discrete	0.24 (0.21)	0.43 (0.28)	* Group [F(1,41) = 6.38, *p* = 0.0155]	DCD–TDC	−0.18	0.07	−2.53	0.0155
	Metronome continuous	0.27 (0.21)	0.42 (0.25)						
rPA	Metronome discrete	22.41 (137.62)	−17.46 (105.00)	Not significant					
	Metronome continuous	39.90 (108.93)	−19.13 (101.07)						

Data are mean (standard deviation). Statistical significant (*) results of the backward repeated mixed model analyses are reported by F(df) = F value, *p*-value. Significant results after post hoc multiple comparison with Tukey–Kramer adjustments are reported. Abbreviations: * statistical significant results of the backwards repeated mixed model analysis, developmental coordination disorder (DCD), number of participants (*n*), relative phase angle (rPA), resultant vector length (RVL), standard error (SE), and typically developing children (TDC).

## Data Availability

All relevant data are presented in this manuscript. The data will be made available upon request from the authors due to privacy and ethical restrictions.
